# Perioperative Management of Paraneoplastic Necrotizing Myopathy in Thyroidectomy: A Case Report

**DOI:** 10.7759/cureus.57351

**Published:** 2024-03-31

**Authors:** Tracy Wong

**Affiliations:** 1 Anaesthesiology and Perioperative Medicine, Singapore General Hospital, Singapore, SGP

**Keywords:** case report, muscular disease, contraindications, anesthesia, malignant hyperthermia

## Abstract

Perioperative management of patients with myopathies can be challenging due to the increased risk of malignant hyperthermia (MH) and anesthesia-induced rhabdomyolysis (AIR). However, currently, there is no evidence regarding the optimal anesthetic management for paraneoplastic necrotizing myopathy (PNM) (total intravenous anesthetic vs. volatile anesthetics). Here, I report a case where anesthesia was administered safely using volatile anesthetics. A 63-year-old female presented with PNM associated with papillary thyroid carcinoma, necessitating urgent thyroidectomy. The patient, previously diagnosed with anti-3-hydroxy-3-methylglutaryl coenzyme A reductase (HMGCR) antibody-associated myopathy, exhibited progressive weakness and dysphagia, prompting suspicion of PNM. The patient's compromised respiratory status, attributed to tracheal compression by a large goiter, necessitated an urgent thyroidectomy. Anesthetic management considerations included the potential effect of HMGCR-M on respiratory muscles and the need for careful planning to mitigate postoperative complications. The patient underwent total thyroidectomy, left central compartment clearance, and tracheostomy. The surgery proceeded uneventfully, with meticulous monitoring and adjustment of anesthetic agents to maintain hemodynamic stability. Postoperatively, the patient recovered well, demonstrating complete resolution of neurological symptoms during a three-month follow-up. The case underscores the importance of recognizing paraneoplastic syndromes in the context of thyroid surgery and highlights potential challenges faced by anesthesiologists. Despite the lack of established safety data for anesthetic drugs in HMGCR-M necrotizing myopathy, the case demonstrates the successful use of sevoflurane and rocuronium.

## Introduction

Certain congenital myopathies, such as central core disease, Evans syndrome, and King-Denborough syndrome, and inherited muscular dystrophies, such as Duchenne’s and Becker’s muscular dystrophies, carry the risks of triggering malignant hyperthermia (MH) and anesthesia-induced rhabdomyolysis (AIR) with inhalational agents [1]. However, there is a lack of literature on the risks of MH and AIR in anti-HMGCR paraneoplastic necrotizing myopathy (PNM).

Anti-HMGCR has been frequently observed to occur in a cohort of patients with statin exposure. However, it can also occur in those who are statin naïve, more commonly in the Asian cohorts. This is the case for the patient mentioned in this case report. Clinically, they have an acute to subacute presentation with marked proximal weakness and dysphagia. Generally, extra-muscular manifestation is absent [2]. An observational study by Allenbach et al. demonstrated that malignancy occurred more frequently (17.3%) in HMGCR-positive patients compared to the general population. No specific type of cancer was predominant [3]. 

PNM is defined by neurological symptoms in the presence of cancer, excluding other known causes [4]. The overall incidence of PNM is less than 1% in patients with cancer. PNM is only diagnosed in patients with cancer after all investigations have ruled out other possible causes [5]. PNM can affect the central nervous system (CNS) and the peripheral nervous system. Generally, CNS PNM is resistant to immunomodulatory treatments. Often, the patients are severely disabled at the time of diagnosis, and no specific treatment is effective. Treatment of the underlying tumor remains the mainstay of management [6].

In this case report, I describe a patient with HMGCR-M, a PNM affecting peripheral muscles, who required thyroidectomy for papillary thyroid carcinoma. The patient provided written informed consent for the publication of this case report.

## Case presentation

**Figure 1:**
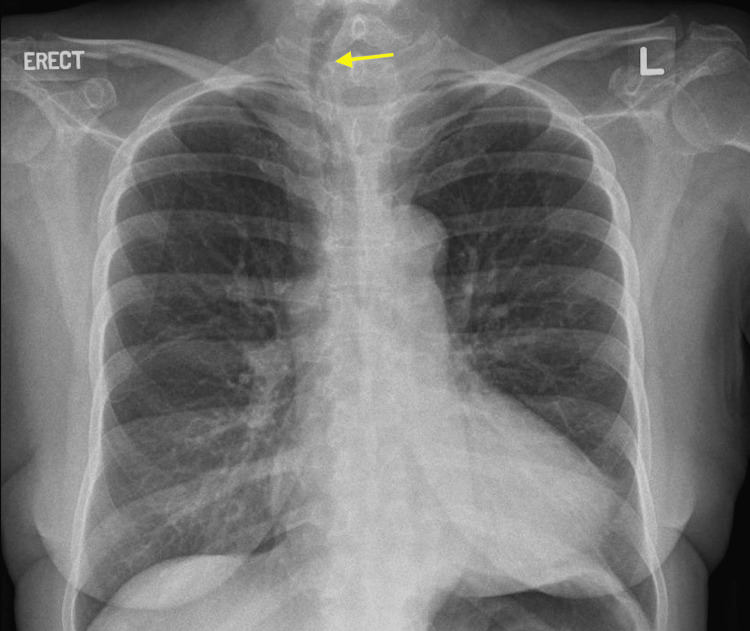
Chest radiograph image showing tracheal deviation toward the right on admission suggestive of an underlying left thyroid goiter.

A 63-year-old chinese female with a medical history of hypertension and type 2 diabetes mellitus presented with progressive weakness in all four limbs and dysphagia four months before admission. She was recently diagnosed with anti-3-hydroxy-3-methylglutaryl coenzyme A reductase (HMGCR) antibody-associated myopathy. Her chest radiograph showed tracheal deviation to the right but no significant narrowing (Figure 1).

Laboratory tests revealed elevated serum levels of creatine kinase (9035 IU/L), myoglobin (3000 ng/mL), and lactate dehydrogenase (75,800 IU/L). The serum alanine and aspartate aminotransferase levels were also elevated. Her lung function test showed a forced expiratory volume (FEV1) of 0.43 L (27% predicted), forced vital capacity (FVC) of 0.46 L (24% predicted), and FEV1/FVC ratio of 0.81 (105% predicted).

**Figure 2:**
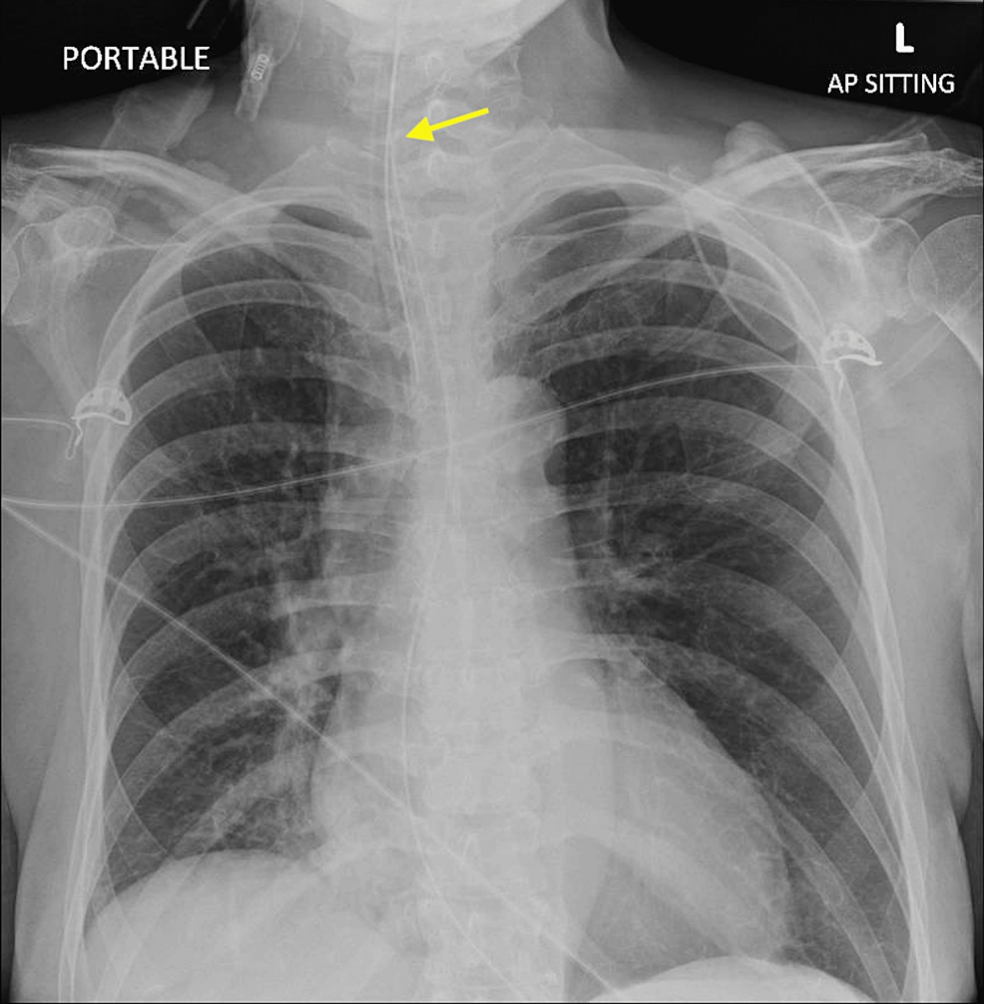
Chest radiograph image showing the positioning of endotracheal and nasogastric feeding tubes post collapse.

On day two of admission, she had worsening breathlessness and experienced pulseless electrical activity (PEA) due to desaturation and decompensation, requiring urgent intubation and one cycle of cardiopulmonary resuscitation before recovering back to sinus rhythm (Figure 2).

**Figure 3:**
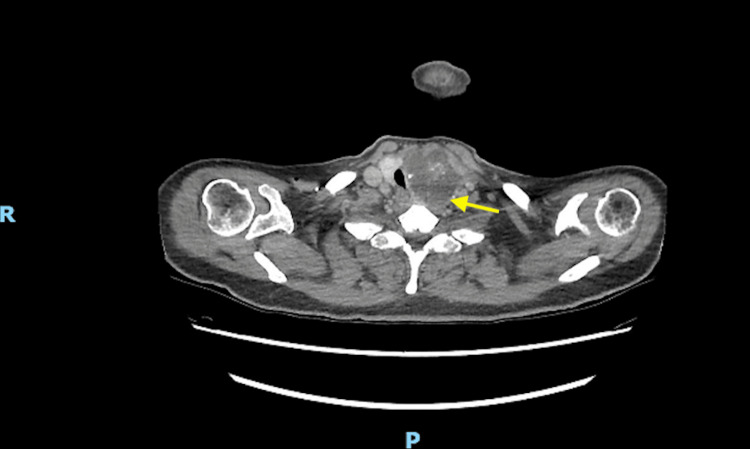
Axial view of the thoracic computed tomography scan showing large, lobulated, irregular, multiloculated mixed cystic/solid left thyroid mass with calcification, extending retrosternally.

**Figure 4:**
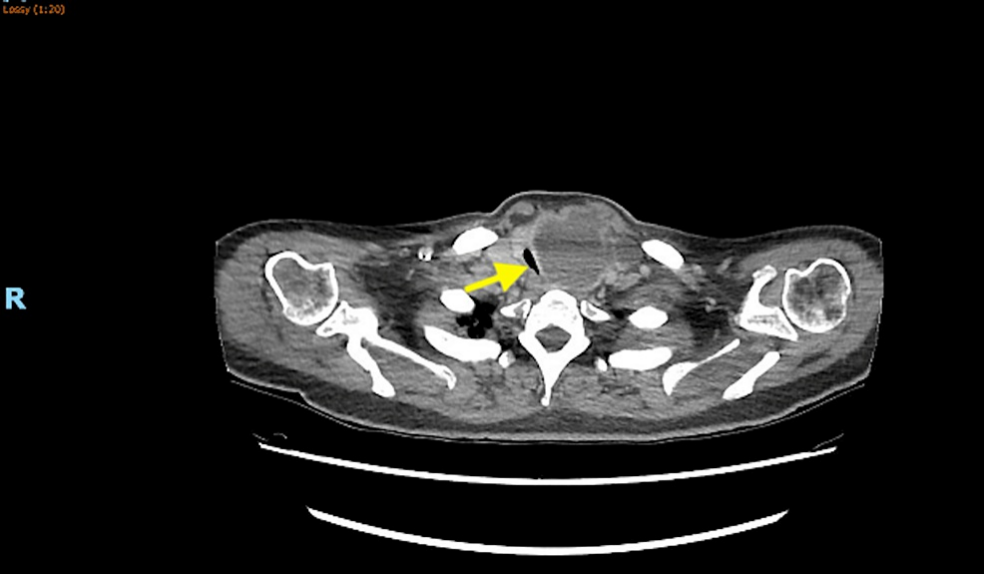
Axial view of the thoracic computed tomography scan showing significant tracheal compression and narrowing.

The patient was admitted to the intensive care unit (ICU) for further evaluation. During her ICU stay, she had a large goiter with retrosternal extension, and significant tracheal compression and narrowing, confirmed using thoracic computed tomography (Figures 3, 4).

Fine-needle aspiration biopsy of the thyroid lesion suggested papillary thyroid carcinoma. Due to her recent diagnosis of anti-HMGCR antibody-associated myopathy (HMGCR-M), potentially causing respiratory muscle weakness, PNM was suspected, which precipitated immunologic factors from papillary thyroid carcinoma. A multidisciplinary team comprising of a neurologist, head and neck surgeon, and anesthesiologist determined that, given her compressive symptoms leading to respiratory collapse, she required an urgent thyroidectomy. She was started on a high dose of prednisolone (60 mg), and the dose of preexisting mycophenolate mofetil was increased from 500 mg to 750 mg BD. 

On day three, after ICU admission, the patient was scheduled for total thyroidectomy, left central compartment clearance, and tracheostomy. Cardiothoracic surgeons were on standby in case a sternotomy was required, and vascular surgeons were on standby in case the goiter was near any vascular structure. She was already intubated with a portex size 6.0 endotracheal tube (ETT). The patient was administered lidocaine (50 mg), midazolam (3 mg), propofol (60 mg) and TCI remifentanil (Minto Model) at 2.5 ng/ml. Train-of-four count (TOFC) was 4, and the train-of-four ratio (TOFR) was 100% at the start of the surgery. After one dose of rocuronium 25 mg was given, the TOFC dropped to 0. 

The preexisting tube was exchanged with a size 6 NIMS ETT with Cook Airway Exchange Catheter using a C-MAC D Blade. The patient was maintained with an air-O_2_ mixture (FiO_2_= 0.45%) on sevoflurane (1.8-2.2%) to obtain a bispectral index (BIS) between 40 and 60 with a BIS monitor. During surgery, TCI remifentanil was titrated between 0.5 and 3 ng/mL to maintain a blood pressure of 95-125/50-65 mmHg and a heart rate of 55-84 beats per minute. Her body temperature was maintained between 36 °C and 36.8 °C, monitored via an esophageal temperature probe. The TOFC returned to 4 after one hour. Further muscle relaxant was not topped up as the NIMS tube was used to monitor the recurrent laryngeal nerve. Remifentanil was used to keep the patient tube-tolerant and immobile during the surgery. The surgery proceeded uneventfully and did not require sternal splitting. The total surgical time was 178 min, with a blood loss of approximately 350 mL and a urine output of 150 mL. During the surgery, 1250 mL of Hartmann’s crystalloid was administered. At the end of the surgery, a large thyroid mass of 6.4 × 4.2 × 4.0 cm was removed. Pathological examination of the mass confirmed a diagnosis of papillary thyroid carcinoma. The patient underwent tracheostomy at the end of the surgery, was transferred to the intensive care unit at the end of the operation, intubated, ventilated, and maintained in synchronized intermittent mandatory ventilation (SIMV) mode until she gradually recovered spontaneous ventilation. At the end of the operation, her TOFC was 4 and the TOFR was 100%; hence, no reversal was required.

On postoperative day one, the patient was weaned off the tracheostomy mask with humidified oxygen at 6 L/min and discharged from the ICU the following morning. On postoperative day 10, the patient was discharged without adverse events. She had a three-month follow-up after discharge, with complete resolution of her neurological symptoms and a well-improved general condition without neurological deficits.

## Discussion

Here, I describe PNM in a patient with an undiagnosed thyroid papillary carcinoma. HMGCR-M can be a clinical manifestation of paraneoplastic syndrome in patients with papillary thyroid carcinoma. However, a definitive link between malignancy and HMGCR-M expression has not been established [7]. The anesthetic management of myopathies can be challenging owing to the associated risks of MH and AIR. However, not all myopathies are associated with MH and AIR. On the contrary, the use of propofol in patients with mitochondrial myopathies may be problematic due to the potential risk of propofol-infusion syndrome. There are no reports of MH and AIR associated with HMGCR-M using volatile anesthetics. Following an individual risk and benefit assessment, a volatile anesthetic was chosen and proven to be well tolerated in this case. Succinylcholine is contraindicated in all patients with myopathies. 

HMGCR-M clinically presents with varying degrees of muscle involvement [8]. In addition to peripheral muscle tissue, anesthesiologists should be aware that myopathies may affect respiratory muscles in severe cases, leading to postoperative respiratory complications, including prolonged mechanical ventilation. This was a concern in the present case; hence, a tracheostomy was performed to support postoperative ventilation. Some myopathies may also cause weakness and fibrous or fatty transformation of cardiac muscles leading to cardiomyopathies, conduction defects, and autonomic instability, resulting in blood pressure fluctuations, arrhythmias, hyperthermia, and diaphoresis [9]. These are important anesthesia-related problems in patients with immune-mediated myopathy that need to be recognized in the perioperative period [9].

To my knowledge, this is the first case report describing the anesthetic implications of paraneoplastic syndrome in a patient presenting with HMGCR-M necrotizing myopathy requiring thyroidectomy [10].

## Conclusions

There are no established data to support the use of specific anesthetic drugs in this context. Clinical trials are unlikely to be performed given that HMGCR-M necrotizing myopathy is a rare disease. I illustrated how sevoflurane and rocuronium could be options for safe anesthesia in patients with this type of myopathy. With sufficient planning and preparation, general anesthesia can be safely administered to patients with HMGCR-M necrotizing myopathy.
